# Endoscopic transcolonic appendiceal detachment – a novel management for appendicitis

**DOI:** 10.1055/a-2851-8381

**Published:** 2026-05-05

**Authors:** Jin Liu, Chaonan Chen, Liang Shang, Xinyu Fan, Junmei Jiang

**Affiliations:** 1 Department of Gastroenterology 34708 Shandong Provincial Hospital Affiliated to Shandong First Medical UniversityJinanChina; 2 Department of Gastrointestinal Surgery 34708 Shandong Provincial Hospital Affiliated to Shandong First Medical UniversityJinanChina

With the development of natural orifice transluminal endoscopic surgery, several groups have reported endoscopic appendectomy for appendiceal lesions 1 2 3 . We describe a less invasive technique, endoscopic transcolonic appendiceal detachment (ETAD). This technique involves meticulous submucosal dissection along the appendiceal base, enabling complete closure of the surgical wound while preserving the structural integrity of the mesoappendix.

A 60-year-old man presented with chronic abdominal discomfort for 1 year. Computed tomography revealed a 0.8 × 0.7 cm ring-enhancing lesion adjacent to the appendiceal orifice ( Fig. 1 **a** ). Endoscopic ultrasound showed a well-demarcated, hypoechoic submucosal lesion measuring 7.3 × 7.1 mm at the appendiceal orifice, and a neuroendocrine tumor could not be excluded ( Fig. 1 **b** ). Because the lesion was small and located at the appendiceal base, standard appendectomy might not have allowed complete resection, whereas cecectomy might have caused greater anatomical alteration. Therefore, ETAD was performed ( Video 1 ).

**Fig. 1:**
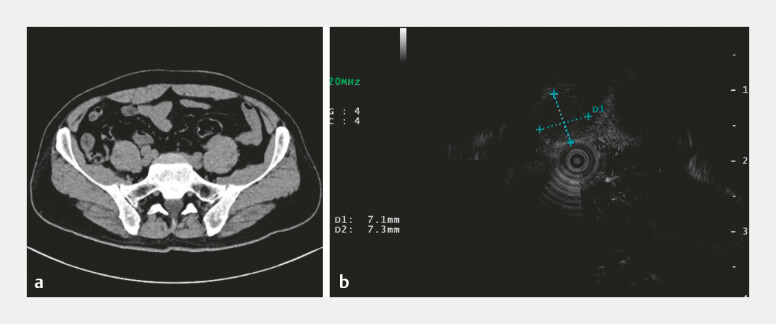
**a** CT scan showed a ring-enhancing lesion (0.8 × 0.7 cm) adjacent to the appendiceal orifice. **b** EUS identified a well-demarcated hypoechoic submucosal lesion at the appendiceal orifice, with heterogeneous echotexture and a cross-sectional diameter of approximately 7.3 × 7.1 mm. CT, computed tomography; EUS, endoscopic ultrasound.

Endoscopic transcolonic appendiceal detachment – a novel management for appendicitis.Video 1

Colonoscopy revealed a submucosal bulge with surface erosion and purulent discharge at the appendiceal orifice ( Fig. 2 **a** ). Lesion margins were marked, followed by submucosal injection and circumferential mucosal incision using a mucosal incision knife ( Fig. 2 **b** ). Stepwise submucosal dissection was then performed ( Fig. 2 **c** ). The endoscope was switched to a double-channel scope; foreign-body forceps retracted the partially dissected appendix while the mucosal incision knife completed detachment. After complete dissection of the mucosal-side root, a deliberate microperforation was created, establishing minimal communication between the intestinal lumen and the peritoneal cavity ( Fig. 2 **d** ). The appendiceal muscularis propria, mesoappendix, and mesenteric-side appendiceal artery were preserved. The wound was closed with titanium clips, and the appendix was dissected free over approximately 3.4 cm ( Fig. 2 **e, f** ).

**Fig. 2:**
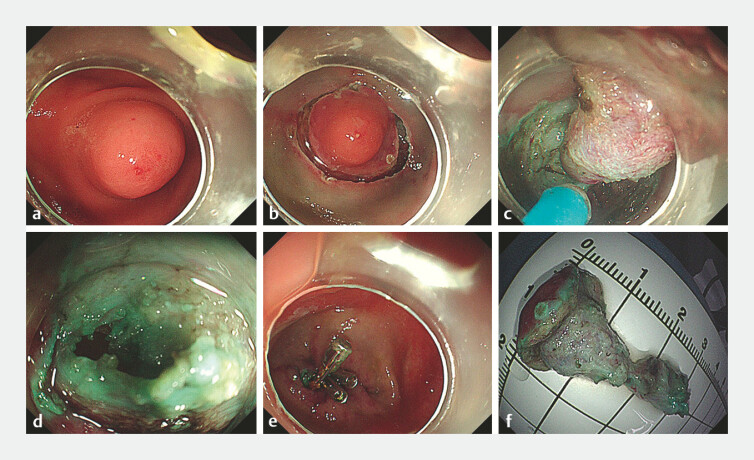
**a** Submucosal bulge with surface erosion and purulent discharge at the appendiceal orifice. **b** Lesion margins were marked, followed by submucosal injection and circumferential mucosal incision. **c** Stepwise submucosal dissection was performed. **d** Deliberate microperforation was created, establishing minimal communication between the intestinal lumen and the peritoneal cavity. **e** Wound closed with titanium clips; appendiceal muscularis propria, mesoappendix, and mesenteric-side appendiceal artery preserved. **f** Appendix dissected free over approximately 3.4 cm.

The total operative duration was 66 minutes. Imipenem was administered for 6 days. Histopathological examination confirmed chronic appendicitis with purulent accumulation within the appendiceal lumen. The total hospital stay was 8 days, and the patient was discharged without obvious complications. At 2-month follow-up colonoscopy, mucosal convergence was observed in the ileocecal region, and the residual clips were removed with a snare. The patient remained asymptomatic.

In conclusion, ETAD may be a feasible minimally invasive option for selected appendiceal lesions.

Endoscopy_UCTN_Code_CCL_1AD_2AJ Endoscopy_UCTN_Code_TTT_1AQ_2AD_3AF

## References

[1] Liu BR Ji T Liu ZH Endoscopic transcecal appendectomy: The first human case report Gastrointestinal Endoscopy 2018 87 311 312 10.1016/j.gie.2017.07.015 28720471

[2] Guo LJ Ye LS Feng YL Endoscopic transcecal appendectomy: A new endotherapy for appendiceal orifice lesions Endoscopy 2022 54 585 590 10.1055/a-1675-2625 34905794 PMC9132730

[3] Chen T Xu AP Lian JJ Transcolonic endoscopic appendectomy: A novel natural orifice transluminal endoscopic surgery (NOTES) technique for the sessile serrated lesions involving the appendiceal orifice Gut 2021 70 1812 1814 10.1136/gutjnl-2020-323018 33483328 PMC8458066

